# Conjunctivitis: Is Astringent Treatment Called For?

**Published:** 1881-07

**Authors:** 


Conjunctivitis: Is Astringent Treatment Called For?—

No. 10 Finsbury Circus, E. C., London,

England, March 8th, 1881.

*Dear Sir:*—The enclosed has been sent to me (alluding to a letter of mine to the *Lancet and Clinic* on "the new departure in the treatment of purulent ophthalmia.")

Will you, in the interest of patients, in some future number of your paper, state that I used my treatment of purulent ophthalmia first in cases of gonorrhœal ophthalmia, and with excellent result. Having succeeded in this worst of purulent ophthalmia, it naturally suggested itself to try it in infants suffering from purulent ophthalmia. I have not found the red oxide of mercury give the least pain and see no cause to give it up.

What I claim as new in my treatment, among others, is the composition of the ointment (mercury, atropine and vaseline), and more especially the mode of applying it, by brushing it well beneath the upper eyelid, and saturating the whole of the conjunctival surface with it.

I shall be glad to give you any further information, and remain, Respectfully yours, C. BADER.

As is well known the idea that *nitrate of silver* is the only remedy in purulency is deep rooted and most persistent; but it has been conclusively demonstrated that this idea will, at least, bear renewing under the modern new thereapeutic light; and certainly no fair-minded man will think of characterizing the reviewers as seeking for novelty rather than truth. No man who has seen any number of conjunctival troubles of any sort, can say he is perfectly satisfied with their present thereapeusis; *hence there is still the possibility of a better.*

It is a pretty true saying that he who sees little has but little to give.

Those who claim to find little of value that is new, simply going on the old saying that what is new is not true, and what is true is not new, can hardly be expected to add to our sum of knowledge.

I am quite prepared to admit that Mr. Bader has obtained most encouraging results, just the same as I am prepared to believe that Becker, of Heidelberg, obtained the most satisfactory results in purulency *without using any remedy upon the conjunctiva.*

Quite recently Prof. Grafe, of Halle, has written a lecture (Volkmann's Sammlung), in the defence of nitrate of silver in purulency, as elaborated by his cousin, Professor von Grafe, characterizing the various plans cropping out, simply as principien-lose Schlendrians, and protesting that he does not believe at all in Leber's view that the basis of action of caustics is *anti-parasitic.* The lecture contains, scientifically, only what is found in all text-books, and is most emphatically obstructive to progress.

As regards conjunctival diseases, I would most earnestly say that so long as under a given plan of treatment, patients may come to us *with no corneal trouble and go away with no cornea at all, there is room for advance.*

It is poor comfort to the general practitioner to inform him that he need not expect to obtain success with astringents and caustics, because he does not know how to apply them. The treatment of conjunctival troubles ought to be made so clear that the wayfaring man, although a fool, need not err therein, and it certainly does not lie in the astringent-caustic plan.

I have had no experience with the *red oxide* employed by Mr. Bader, but accept what his experience shows, *that it does not cause pain*, even though classed as an escharotic in the materia medica. His combination of salve is certainly irrational, for atropia acts as a *dilator* of blood-vessels, and is improper, and he is welcome to any *originality* in his mixture. I am more and more convinced that for conjunctival troubles, the salve *first devised* and *used by myself*, consisting of the amorphorus yellow oxide of four or five grains to the half-ounce of vaseline, is altogether the most efficient remedy yet used, being free from irritation and danger. W. W. SEELY, A.M., M.D.

—*Lancet and Clinic.*

